# Water quality assessment and evaluation of human health risk of drinking water from source to point of use at Thulamela municipality, Limpopo Province

**DOI:** 10.1038/s41598-022-10092-4

**Published:** 2022-04-11

**Authors:** N. Luvhimbi, T. G. Tshitangano, J. T. Mabunda, F. C. Olaniyi, J. N. Edokpayi

**Affiliations:** 1 Department of Public Health, School of Health Sciences, University of Venda, Thohoyandou, 0950 South Africa; 2 Department of Hydrology and Water Resources, School of Environmental Sciences, University of Venda, Thohoyandou, 0950 South Africa

**Keywords:** Environmental sciences, Risk factors

## Abstract

Water quality has been linked to health outcomes across the world. This study evaluated the physico-chemical and bacteriological quality of drinking water supplied by the municipality from source to the point of use at Thulamela municipality, Limpopo Province, South Africa; assessed the community practices regarding collection and storage of water and determined the human health risks associated with consumption of the water. Assessment of water quality was carried out on 114 samples. Questionnaires were used to determine the community’s practices of water transportation from source to the point-of-use and storage activities. Many of the households reported constant water supply interruptions and the majority (92.2%) do not treat their water before use. While *E. coli and* total coliform were not detected in the water samples at source (dam), most of the samples from the street taps and at the point of use (household storage containers) were found to be contaminated with high levels of *E. coli* and total coliform. The levels of *E. coli and* total coliform detected during the wet season were higher than the levels detected during the dry season. Trace metals’ levels in the drinking water samples were within permissible range of both the South African National Standards and World Health Organisation. The calculated non-carcinogenic effects using hazard quotient toxicity potential and cumulative hazard index of drinking water through ingestion and dermal pathways were less than unity, implying that consumption of the water could pose no significant non-carcinogenic health risk. Intermittent interruption in municipal water supply and certain water transportation and storage practices by community members increase the risk of water contamination. We recommend a more consistent supply of treated municipal water in Limpopo province and training of residents on hygienic practices of transportation and storage of drinking water from the source to the point of use.

## Introduction

Water is among the major essential resources for the sustenance of humans, agriculture and industry. Social and economic progress are based and sustained upon this pre-eminent resource^1^. Availability and easy access to safe and quality water is a fundamental human right^2^ and availability of clean water and sanitation for all has been listed as one of the goals to be achieved by the year 2030 for sustainable development by the United Nations General Assembly (UNGA)^3^.

The physical, chemical, biological and aesthetic properties of water are the parameters used to describe its quality and determine its capability for a variety of uses including the protection of human health and the aquatic ecosystem. Most of these properties are influenced by constituents that are either dissolved or suspended in water and water quality can be influenced by both natural processes and human activities^4,5^. The capacity of a population to safeguard sustainable access to adequate quantities and acceptable quality of water for sustaining livelihoods of human well-being and socioeconomic growth; as well as ensuring protection against pollution and water related disasters; and for conserving ecosystems in a climate of peace and political balance is regarded to as water security^6^.

Although the world’s multitudes have access to water, in numerous places, the available water is seldom safe for human drinking and not obtainable in sufficient quantities to meet basic health needs^7^. The World Health Organization (WHO) estimated that about 1.1 billion people globally drink unsafe water and most diarrheal diseases in the world (88%) is attributed to unsafe water, poor sanitation and unhygienic practices. In addition, the water supply sector is facing enormous challenges due to climate change, global warming and urbanization. Insufficient quantity and poor quality of water have serious impact on sustainable development, especially in developing countries^8^.

The quality of water supplied by the municipality is to be measured against the national standards for drinking water developed by the federal governments and other relevant bodies^9^. These standards considered some attributes to be of primary importance to the quality of drinking water, while others are considered to be of secondary importance. Generally, the guidelines for drinking water quality recommend that faecal indicator bacteria (FIB), especially *Escherichia coli* (*E. coli*) or thermo tolerant coliform (TTC), should not be found in any 100 mL of drinking water sample^8^.

Despite the availability of these standards and guidelines, numerous WHO and United Nations International Children Emergency Fund (UNICEF) reports have documented faecal contamination of drinking water sources, including enhanced sources of drinking water like the pipe water, especially in low-income countries^10^. Water-related diseases remain the primary cause of a high mortality rate for children under the age of five years worldwide. These problems are specifically seen in rural areas of developing countries. In addition, emerging contaminants and disinfection by-products have been associated with chronic health problems for people in both developed and developing countries^11^. Efforts by governmental and non-governmental organizations to ensure water security and safety in recent years have failed in many areas due to a lack of sustainability of water supply infrastructures^12^.

Water quality, especially regarding the microbiological content, can be compromised during collection, transport, and home storage. Possible sources of drinking water contamination are open field defecation, animal wastes, economic activities (agricultural, industrial and businesses), wastes from residential areas as well as flooding. Any water source, especially is vulnerable to such contamination^13^. Thus, access to a safe source alone does not ensure the quality of water that is consumed, and a good water source alone does not automatically translate to full health benefits in the absence of improved water storage and sanitation^14^. In developing countries, it has been observed that drinking-water frequently becomes re-contaminated following its collection and during storage in homes^15^.

Previous studies in developing countries have identified a progressive contamination of drinking water samples with *E. coli* and total coliforms from source to the point of use in the households, especially as a result of using dirty containers for collection and storage processes^16–18^. Also, the type of water treatment method employed at household levels, the type of container used to store drinking water, the number of days of water storage, inadequate knowledge and a lack of personal and domestic hygiene have all been linked with levels of water contamination in households^19,20^.

In South Africa, many communities have access to treated water supplied by the government. However, the water is more likely to be piped into individual households in the urban than rural areas. In many rural communities, the water is provided through the street taps and residents have to collect from those taps and transport the water to their households. Also, water supply interruptions are frequently experienced in rural communities, hence, the need for long-term water storage. A previous study of water quality in South Africa reported better quality of water at source than the water samples obtained from the household storage containers, showing that water could be contaminated in the process of transporting it from source to the point of use^21^.

This study was conducted in a rural community at Thulamela Municipality, Limpopo province, South Africa, to describe the community’s drinking water handling practices from source to the point of use in the households and evaluate the quality of the water from source (the reservoir), main distribution systems (street taps), yard connections (household taps) and at the point of use (household storage containers). Water quality assessment was done by assessing the microbial contamination and trace metal concentrations, and the possible health risks due to exposure of humans to the harmful pathogens and trace metals in the drinking water were determined.

## Methods

### Study area

**Figure 1:**
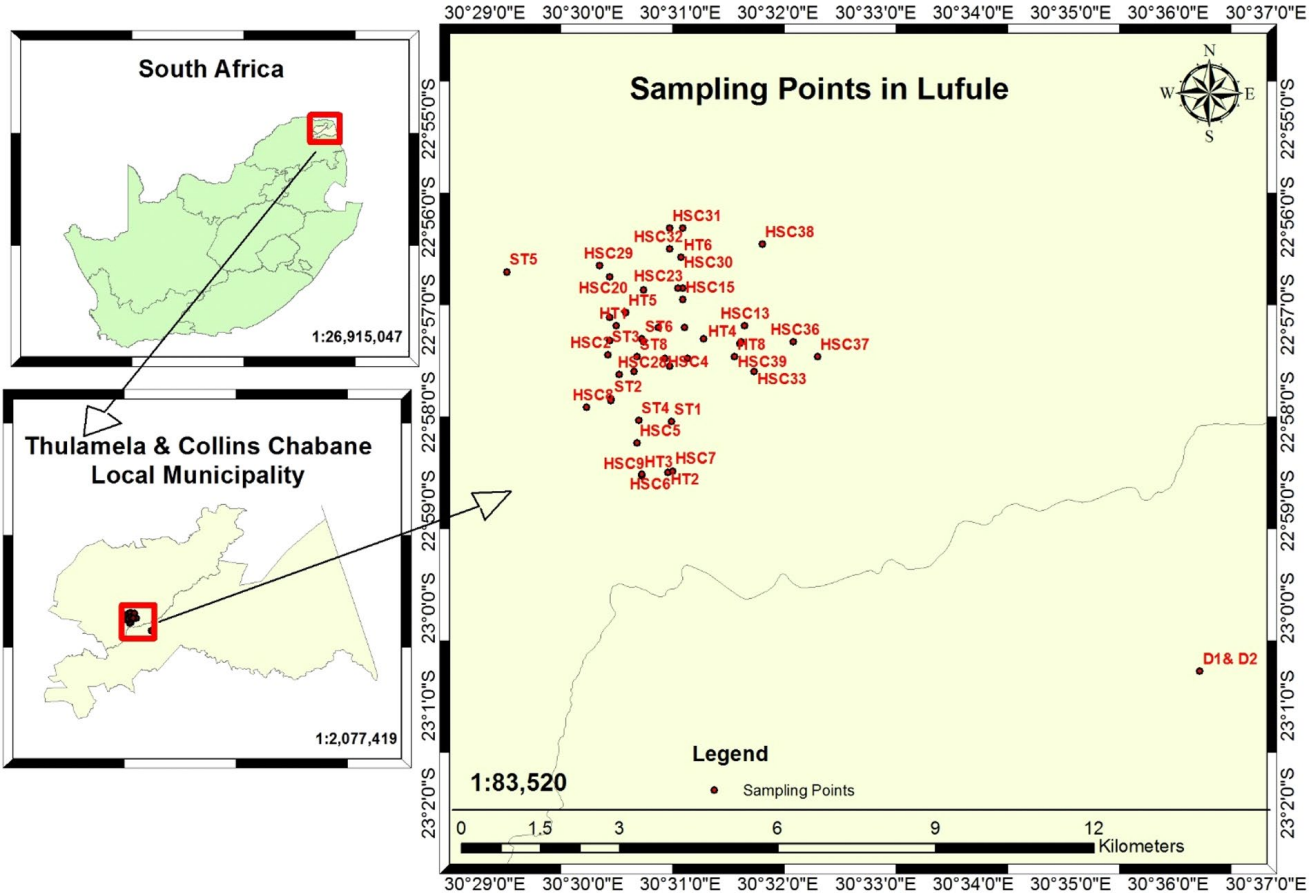
Map of the study area showing water samples’ collection areas.

The study was conducted at Lufule village in Thulamela municipality, Limpopo Province, South Africa. The municipality is situated in the eastern subtropical region of the province. The province is generally hot and humid and it receives much of its rainfall during summer (October–March)^22^. Lufule village is made up of 386 households and a total population of 1, 617 residents^23^. The study area includes Nandoni Dam (main reservoir) which acquires its raw water from Luvuvhu river that flows through Mutoti and Ha-Budeli villages just a few kilometers away from Thohoyandou town. Nandoni dam is where purification process takes place to ensure that the water meets the standards set for drinking water. This dam is the main source of water around the municipality, and it is the one which supplies water to selected areas around the dam, including Lufule village. Water samples for analysis were collected from the dam (D), street taps (ST), household taps (HT) and household storage containers (HSC) (Fig. 1).

### Research design

This study adopted a quantitative design comprising of field survey and water analysis.

### Field survey

The survey was done to identify the selected households and their shared source of drinking water (street taps). The village was divided into 10 quadrants for sampling purposes. From each quadrant, 6 households were randomly selected where questionnaires were distributed and household water samples were also collected for analysis.

### Quantitative data collection

A structured interviewer-administered questionnaire was employed for data collection in the selected households. The population of Lufule village residents aged 15–69 years is 1, 026 (Census, 2011). About 10% of the adult population (~ 103) was selected to complete the questionnaires to represent the entire population. However, a total of 120 questionnaires were distributed, to take care of those which might be lacking vital information and therefore would not qualify to be analysed. Adults between the ages of 18 and 69 years were randomly selected to complete the questionnaire which includes questions concerning demographic and socio-economic statuses of the respondents, water use practices, sanitation, hygiene practices as well as perception of water quality and health. The face validity of the instrument was ensured by experts in the Department of Public Health, University of Venda, who reviewed questionnaire and confirmed that the items measure the concepts of interest relevant to the study^24^. Respondents were given time to go through the questionnaire and the researcher was present to clear any misunderstanding that may arise.

### Water sampling

Permission to collect water samples from the reservoir tank at the Nandoni water treatment plant and households was obtained from the plant manager and the households’ heads respectively. Two sampling sites were identified at the dam, from where a water sample each was collected during the dry and the wet season. Similarly, 8 sampling sites were identified from the street and household taps, while 60 sampling sites were targeted for the household storage containers. However, only 39 household sites were accessible for sample collection, due to unavailability of the residents at the times of the researcher’s visit. Thus, water samples were collected from a total of 57 sites. Samples were collected from each of the sites during the dry (12th–20th April, 2019) and wet seasons (9th–12th December, 2019) between the hours of 08h00 and 14h30. A total of 114 samples were collected during the sampling period: 4 from the reservoir, 16 from street taps, 16 from household taps and 78 from households’ storage systems. Water samples were collected in 500 mL sterile polyethylene bottles. After collection, the containers were transported to the laboratory on ice in a cooler box. Each of the samples was tested for physico-chemical parameters, microbial parameters and trace metals’ concentration.

### Physicochemical parameters’ analysis

Onsite analysis of temperature, pH, Electrical conductivity (EC) and Total Dissolved Solids (TDS) were performed immediately after sampling using a multimeter (model HI “HANNA” instruments), following the standards protocols and methods of American Public Health Association (APHA)^25^. The instrument was calibrated in accordance with the manufacturer’s guideline before taking the measurements. The value of each sample was taken after submerging the probe in the water and held for a couple of minutes to achieve a reliable reading. After measurement of each sample, the probe was rinsed with de-ionized water to avoid cross contamination among different samples.

### ICP-OES and ICP-MS analyses of major and trace elements

An inductively coupled plasma optical emission spectrophotometer (ICP-OES) was used to analyse the major metals (Calcium (Ca), Sodium (Na), Potassium (K) and Magnesium (Mg)) in the water samples while inductively coupled plasma mass spectrophotometer (ICP-MS) was used to analyze the trace metals. The instrument was standardized with a multi-element calibration standard IV for ICP for Copper (Cu), Manganese (Mn), Iron (Fe), Chromium (Cr), Cadmium (Cd), Arsenic (As), Nickel (Ni), Zinc (Zn), Lead (Pb) and Cobalt (Co) and analytical precision was checked by frequently analysing the standards as well as blanks. ICP multi Standard solution of 1000 ppm for K, Ca, Mg and Na was prepared with NH_4_OAC for analysis to verify the accuracy of the calibration of the instrument and quantification of selected metals before sample analysis, as well as throughout the analysis to monitor drift.

### Microbiological water quality analysis

Analysis of microbial parameters was conducted within 6 h of collection as recommended by APHA^25^. Viable Total coliform and *E. coli* were quantified in each sample using the IDEXX technique approved by the United States Environmental Protection Agency (USEPA). Colilert media was added to 100 mL sample and mixed until dissolved completely. The solution was poured into an IDEXX Quanti-Tray/2000 and sealed using the Quanti-Tray sealer^26^. The samples were incubated at 35 °C for 24 h. Trays were scanned using a fluorescent UV lamp to count fluorescent wells positive for *E. coli* concentration and counted with the most probable number (MPN) table provided by the manufacturer^27^.

### Health risk assessment

Risk assessment have been estimated for ingestion and dermal pathways. Exposure pathway to water for ingestion and dermal routes are calculated using Eqs. (1) and (2) below:

1 $$ {\text{Exp}}_{{{\text{ing}}}} = \frac{{IR \times C_{water} \times EF \times ED}}{AT \times BW} $$

2 $$ {\text{Exp}}_{{{\text{derm}}}} = \frac{{C_{water} \times SA \times ET \times EF \times ED \times CF \times K_{p} }}{AT \times BW} $$

where Exp_ing_: exposure dose through ingestion of water (mg/kg/day); BW: average body weight (70 kg for adults; 15 kg for children); Exp_derm_: exposure dose through dermal absorption (mg/kg/day); C_water_: average concentration of the estimated metals in water (μg/L); IR: ingestion rate in this study (2.0 L/day for adults; 1.0 L/day for children); ED: exposure duration (70 years for adults; and 6 years for children);AT: averaging time (25,550 days for an adult; 2190 days for a child); EF: exposure frequency (365 days/year) SA: exposed skin area (18.000 cm^2^ for adults; 6600 cm^2^ for children); K_p_: dermal permeability coefficient in water, (cm/h), 0.001 for Cu, Mn, Fe and Cd, while 0.0006 for Zn; 0.002 for Cr and 0.004 for Pb; ET: exposure time (0.58 h/ day for adults; 1 h/day for children) and CF: unit conversion factor (0.001 L/cm^3^)^28^.

The hazard quotient (HQ) of non-carcinogenic risk by ingestion pathway can be determined by Eq. (3)

3 $$ {\text{HQ}}_{{\text{ing/derm}}} = \frac{{EXP_{ing/derm} }}{{RfD_{ing/derm} }} $$

where RfD_ing_ is ingestion toxicity reference dose (mg/kg/day). An HQ under 1 is assumed to be safe and taken as significant non-carcinogenic, but HQ value above 1 may indicate a major potential health concern associated with over-exposure of humans to the contaminants^28^.

The total non-carcinogenic risk is represented by hazard index (HI). HI < 1 means the non-carcinogenic risk is acceptable, while HI > 1 indicates the risk is beyond the acceptable level^29^. The HI of a given pollutant through multiple pathways can be calculated by summing the hazard quotients by Eq. (4) below.

4 $$ {\text{HI}} = \mathop \sum \limits_{i = 1}^{n} HQ_{ing/derm} $$

Carcinogenic risks for ingestion pathway is calculated by Eq. (5). For the selected metals in the study, carcinogenic risk (CR_ing_) can be defined as the probability that an individual will develop cancer during his lifetime due to exposure under specific scenarios^30^.

5 $$ {\text{CR}}_{{{\text{ing}}}} = \frac{{EXP_{ing} }}{{SF_{ing} }} $$

where CRing is carcinogenic risk via ingestion route and SF^ing^ is the carcinogenic slope factor.

### Data analysis

Data obtained from the survey were analysed using Microsoft Excel and presented as descriptive statistics in the form of tables and graphs. The experimental data obtained was compared with the South African National Standards (SANS)^31^ and Department of Water Affairs and Forestry (DWAF)^32^ guidelines for domestic water use.

### Ethics approval and consent to participate

The ethical clearance for this study was granted by the University of Venda Health, Safety and Research Ethics’ Committee (SHS/19/PH/14/1104). Permission to conduct the study was obtained from the Department of Water affairs, Limpopo province, Vhembe district Municipality and the selected households. Respondents were duly informed about the study and informed consent was obtained from all of them. The basic ethical principles of voluntary participation, informed consent, anonymity and confidentiality of respondents were duly complied with during data collection, analysis and reporting.

### Consent for publication

Not applicable.

## Results

### Socio-demographic characteristics of respondents

A total of 120 questionnaires were distributed but only 115 were completed, making a good response rate of 95%. The socio-demographic characteristics of the respondents are presented in Table 1.

**Table 1:** Socio- demographic characteristics of the respondents.

Socio-demographic characteristics		Frequency (n)	Percentage (%)
Gender	Male	78	67.8
	Female	37	32.2
Age (in years)	18–24	16	13.9
	25–34	40	34.8
	35–44	11	9.6
	45–54	19	16.5
	55–64	9	7.8
	> 64	20	17.4
Level of education	Primary school	7	6.1
	Secondary school	34	29.6
	Matriculation	53	46.0
	Undergraduate	7	6.1
	Postgraduate	14	12.2
Employment status	Permanent employment	25	21.7
	Temporary employment	4	3.5
	Self-employment	49	42.6
	Grant holder	29	25.2
	Others	8	7.0

### Household water supply

Many households (68.7%) had their primary water source from the municipality piped into their yards, but only 5.2% have the water flowing within their houses. The others have to fetch water at their neighbours’ yards or use the public taps on the streets. When the primary water supply is interrupted (i.e. when there is no water flowing through the pipes within the houses, yards or the public taps due to water rationing activities by the municipality, leakage of water distribution pipes, vandalization of pipes during road maintenance, etc.), the interruption usually lasts between a week or two, during which the respondents resort to other alternative sources. A return trip to the secondary source of water usually takes between 10 and 30 min for more than half of the respondents (53.0%) (Table 2).

**Table 2:** Water supply in the household.

Variables		Frequency (n)	Percentage (%)
Primary source of drinking water	Piped into the house	6	5.2
	Piped into the yard	79	68.7
	Neighbours’ pipe	22	19.1
	Public tap or standpipe	6	5.2
	Other	2	1.7
Duration of water supply interruption	1 week	68	59.2
	2 weeks	36	31.3
	Month	2	1.7
	2 months	3	2.6
	3 months	6	5.2
Secondary source of water during interruptions	Municipality treated source	4	3.5
	Communal tap	58	50.4
	Private boreholes	24	20.9
	Other	29	25.2
Time taken to get water from the secondary water source and return in one trip	10–30 min	61	53.0
	35 min–1 h	38	33.0
	1 h 30 min–2 h	11	9.6
	2 h 30 min–3 h	4	3.5
	3 h or more	1	0.9

### Water storage and treatment practices at the household

**Figure 2:**
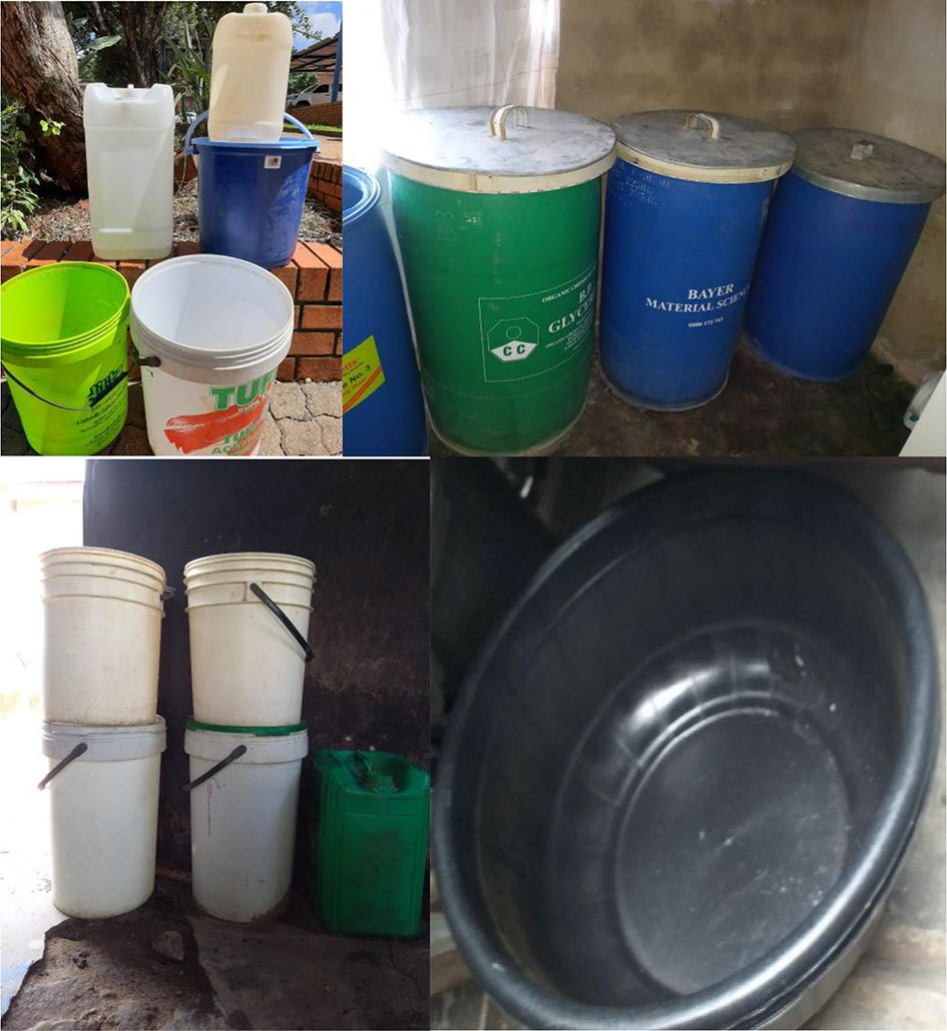
Examples of household water storage containers, some with lids and others without lids (photo from fieldwork).

Household water was most frequently stored in plastic buckets (n = 78, 67.8%), but ceramic vessels, metal buckets and other containers are also used for water storage (Fig. 2). Most households reported that their drinking water containers were covered (n = 111, 96.5%). More than half (53.9%) of the respondents used cups with handles to collect water from the storage containers whereas 37.4% used cups with no handles. Only 7.8% households reported that they treat their water before use mainly by boiling. Approximately 82.6% of respondent are of the opinion that one cannot get sick from drinking water and only 17.4% knew the risks that come with untreated water, and cited diarrhoea, schistosomiasis, cholera, fever, vomiting, ear infections, malnutrition, rash, flu and malaria as specific illnesses associated with water. Despite these perceptions, the majority (76.5%) were satisfied with their current water source. The few (23.5%) who were not satisfied cited poor quality, uncleanness, cloudiness, bad odour and taste in the water as reasons for their dissatisfaction (Table 3).

**Table 3:** Water storage and treatment practices at the household.

Variables		Frequency (n)	Percentage (%)
Do you usually treat your water before you drink?	Yes	9	7.8
	No	106	92.2
Which method of treatment do you usually use to make your water safe to drink?	Solar disinfection	2	1.7
	Water filter (ceramic/sand/etc.)	3	2.6
	Bleach/chlorine	2	1.7
	Boiling	2	1.7
Where do you store your drinking water?	Ceramic vessels	24	20.9
	Metal buckets	5	4.3
	Plastic buckets	78	67.8
	Jerrycan	3	2.6
	Pans	3	2.6
	Water tank	2	1.7
How long does water stay in the storage container?	Less than a week	101	87.8
	1–2 weeks	8	7.0
	Less than a month	6	5.2
Are your storage vessels covered?	No	4	3.5
	Yes	111	96.5
What do you use to get the water from the storage container?	Pour directly	10	8.7
	Use cup with handle	62	53.9
	Use cup with no handle	43	37.4
Do you think that you can get sick from the water you use?	No	95	82.6
	Yes	20	17.4
Are you satisfied with the quality of water you use?	No	27	23.5
	Yes	88	76.5

### Sanitation practices at the household level

More than half of the respondents (67%) use pit toilets, whereas only 26.1% use the flush to septic tank system, most of the toilets (93.9%) have a concrete floor. About 76.5% of households do not have designated place to wash their hands, however, all respondents indicated that they always wash their hands with soap or any of its other alternatives before preparing meals and after using the toilet (Table 4).

**Table 4:** Sanitation practices at the household level.

Variables		Frequency (n)	Percentage (%)
What kind of toilet facility do you and other members of your household usually use?	No facility/bush/fields	3	2.6
	Pit latrine	77	67.0
	Improved pit latrine without flush	3	2.6
	Flush to piped sewer system	2	1.7
	Flush to pit (septic) tank	30	26.1
Does the toilet/latrine you use have a concrete floor	No	7	6.1
	Yes	108	93.9
How many households use this toilet facility?	1–2	53	46.1
	3–5	6	5.2
	6–8	11	9.6
	9–10	22	19.1
	12–15	22	19.1
	16–20	1	0.9
Is there a designated place to wash hands by this toilet?	No	88	76.5
	Yes	27	23.5
How often do you wash your hands after using the toilet?	Always	115	100.0
How often do you use toilet paper?	Always	115	100.0
How often do you wash your hands before preparing food?	Always	115	100.0
How often do you use soap when you wash your hands? (soap can include ash, sand or the use of hand sanitizer gel/ cream)	Always	115	100.0

### Water samples analysis

The water samples analyses comprise of microbial analysis, physico-chemical analysis and trace metals' parameters.

#### Microbial analysis

The samples from the reservoir during dry and wet season had 0 MPN/100 mL of total coliform and *E. coli* and were within the recommended limits of WHO and SANS for drinking water. During the wet season, seven out of the eight water samples collected from the street taps were contaminated with total coliform, while four of the samples taken from the same source were contaminated with total coliform during the dry season. Water samples from street taps 3 and 7 (ST 3 and ST7) were contaminated with total coliform during both seasons, however, the total coliform counts during the wet season were more than the counts during the dry season. None of the samples was contaminated with *E. coli* during the dry season, however, 2 samples from the street taps (ST3 & ST6) were found to be contaminated with *E. coli* during the wet season. Samples from household taps showed a similar trend with the street taps—with all samples being contaminated with total coliform during the wet season. Though 7 of the 8 samples taken from the household taps were contaminated with total coliform during the dry season, the samples from the same sources showed a higher level of total coliform in the wet season, with almost all the samples showing contamination at maximum detection levels of more than 2000 MPN/100 mL, except one sample (HT8) which showed a higher level of contamination with total coliform during the dry compared with the wet season. Only one sample (HT4) was found to be contaminated with *E. coli* during both dry and wet season. This shows that total coliform contamination levels are higher during the wet season than the dry season (Table 5).

**Table 5:** Total coliform and *E. coli* levels in water samples from the reservoir, street taps and household taps.

Sample ID	Total coliform (MPN/100 mL)		*E. coli* (MPN/100 mL)	
	Dry season	Wet season	Dry season	Wet season
**Reservoir/ Dam(D)**				
D1	0	0	0	0
D2	0	0	0	0
**Street taps (ST)**				
ST 1	0	> 2000	0	0
ST 2	0	144.8	0	0
ST 3	132.4	> 2000	0	1.0
ST 4	0	0	0	0
ST 5	123.9	123.9	0	0
ST 6	770.1	770.1	0	1.0
ST 7	221.3	> 2000	0	0
ST 8	146.1	62.9	0	0
**Household taps (HT)**				
HT 1	1.0	> 2000	0	0
HT 2	0	410.6	0	0
HT 3	3.0	> 2000	0	0
HT 4	78.7	200.5	1.0	1.0
HT 5	157.5	> 2000	0	0
HT 6	3.0	> 2000	0	0
HT 7	15.8	> 2000	0	0
HT 8	> 2000	307.6	0	0
**Reference values**				
SANS^31^	10	10	0	0
WHO^8^	0	0	0	0

Water samples from household storage containers (HSC) showed a higher level of total coliform during the wet season than the dry season and more samples were contaminated with *E. coli* during the wet season also (Table 6). A higher level of contamination was recorded for the HSCs compared to the street and household taps.

**Table 6:** Total coliform and *E. coli* levels for household storage containers.

Household storage containers (HSC)	Total coliform		*E. coli*	
	Dry season	Wet season	Dry season	Wet season
HSC 1	> 2000	NA	0	NA
HSC 2	31.5	> 2000	0	0
HSC 3	> 2000	> 2000	1.0	1.0
HSC 4	> 2000	307.6	0	0
HSC 5	172.2	> 2000	0	0
HSC 6	224.7	1553.1	0	0
HSC 7	> 2000	579.4	0	0
HSC 8	886.4	> 2000	2.0	0
HSC 9	1299.7	48.2	0	0
HSC 10	> 2000	> 2000	0	0
HSC 11	1553.1	39.5	0	0
HSC 12	> 2000	48.2	0	0
HSC 13	47.1	62.9	0	0
HSC 14	1.0	33.6	0	0
HSC 15	38.4	NA	0	NA
HSC 16	> 2000	> 2000	0	0
HSC 17	> 2000	> 2000	0	1.4
HSC 18	1732.9	1119.9	0	0
HSC 19	> 2000	> 2000	0	0
HSC 20	> 2000	> 2000	0	0
HSC 21	245.2	135.5	0	0
HSC 22	0	57.4	0	2.0
HSC 23	0	NA	0	NA
HSC 24	1.0	146.1	0	0
HSC 25	8.5	133.0	0	0
HSC 26	7.5	57.1	0	0
HSC 27	0	146.1	0	0
HSC 28	2.0	NA	0	NA
HSC 29	0	> 2000	0	0
HSC 30	0	95.7	0	0
HSC 31	0	> 2000	0	0
HSC 32	0	> 2000	0	0
HSC 33	206.4	> 2000	0	0
HSC 34	1119.9	> 2000	0	1.0
HSC 35	261.3	> 2000	0	0
HSC 36	15.8	> 2000	0	0
HSC 37	229.4	> 2000	0	0
HSC 38	15.8	NA	1.0	NA
HSC 39	> 2000	229.4	0	0
**Reference Values**				
SANS^31^	10	10	0	0
WHO^8^	0	0	0	0

NA = not available.

#### Physico-chemical analysis

**Figure 3:**
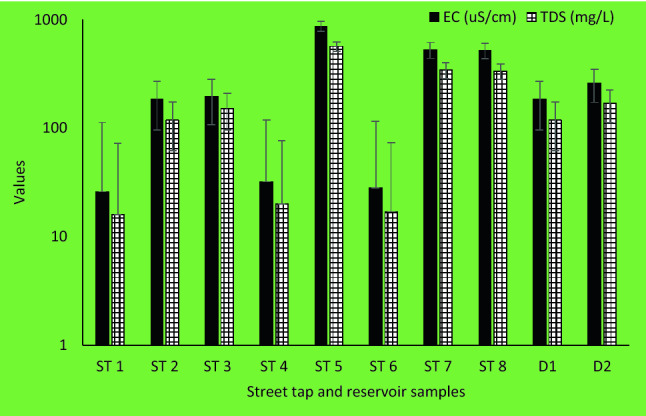
EC and TDS levels for the street taps and reservoir samples.

In the reservoir samples, the pH value ranged from 8.37 to 8.45, EC ranged between 183 and 259 µS/cm whereas TDS varied between 118 and 168 mg/L. Similarly, in the street tap samples, pH value ranged from 7.28 and 9.33, EC ranged between 26 and 867 µS/cm whereas TDS varied between 16 and 562 mg/L (Fig. 3).

**Figure 4:**
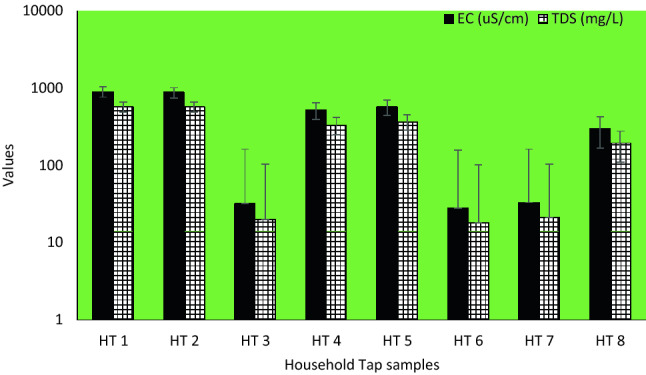
EC and TDS levels for household taps.

In the household taps, pH value ranged from 7.70–9.98, EC range between 28–895 µS/cm and TDS varied between 18 and 572 mg/L (Fig. 4).

**Figure 5:**
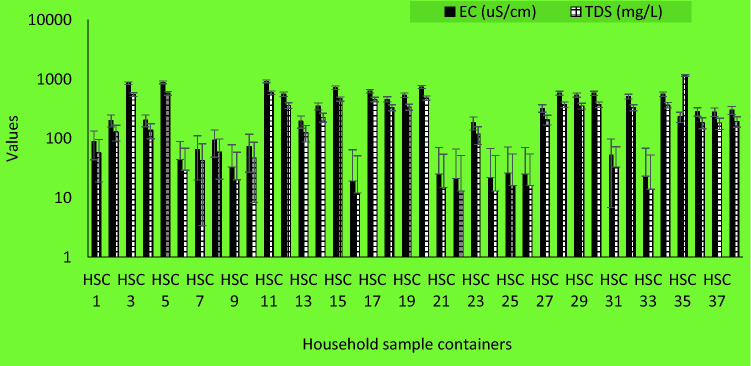
EC and TDS levels for household storage container samples.

In household storage container samples, the pH value ranges from 7.67–9.77, EC ranged between 19–903 µS/cm and TDS values ranged from 12–1148 mg/L (Fig. 5).

### Analysis of cations and trace metals in water

To detect the cations’ and trace metals’ concentrations in the water samples, representative samples from each of the sources were selected for analysis. The concentration of Calcium ranged between 2.14 and 31.65 mg/L, Potassium concentration ranged from 0.14 to 1.85 mg/L, Magnesium concentration varied from 1.32 to 16.59 mg/L, Sodium ranged from 0.18 to 12.96 mg/L (Table 7).

**Table 7:** Cations indicators in water.

Metals (mg/L)	Ca	K	Mg	Na
LOD	0.1	0.1	0.1	0.1
% recovery	95	111	96	95
D1	11.81	*BDL	6.75	7.61
D2	11.64	1.85	6.45	9.44
ST 1	20.95	*BDL	11.26	7.09
ST 3	26.99	0.31	14.22	11.12
ST 4	5.05	*BDL	2.50	0.56
ST 5	31.65	0.49	16.59	12.96
ST 6	3.41	0.15	1.59	2.57
ST 7	2.84	0.16	1.56	2.54
HT 2	30.99	0.29	16.30	12.62
HT 4	25.66	*BDL	13.57	8.83
HT 8	11.54	1.83	6.44	9.27
HSC 9	2.14	0.14	1.32	2.42
HSC 28	3.27	*BDL	1.76	0.18
HSC 30	16.24	*BDL	8.72	5.09
HSC 33	26.55	*BDL	13.85	8.95
DWAF ^32^	32	50	30	100

LOD- Limit of detection; *BDL -below detection limit.

### Trace metals’ analysis

The minimum and maximum concentrations of trace metals (Al, Mn, Fe, Co, Ni, Cu, Zn, As and Pb) present in water samples from selected street taps, household taps and household storage containers are presented in Table 8.

**Table 8:** Concentration of trace metals in water.

Metals (µg/L)	Al	Mn	Fe	Co	Ni	Cu	Zn	As	Pb
LOD	0.31	0.14	0.31	0.01	0.10	0.19	0.32	0.02	0.02
% Accuracy QC	99	101	108	96	93	94	96	92	102
D1	13.49	4.09	19.67	0.03	0.35	2.69	4.14	0.14	0.12
D2	12.65	4.44	21.45	0.04	0.38	1.51	3.49	0.13	0.11
ST 1	2.97	1.27	2.28	0.02	0.17	2.24	23.16	0.03	0.06
ST 3	2.12	1.22	1.16	*BDL	0.32	1.67	8.99	*BDL	0.04
ST 4	1.36	1.25	5.41	0.02	0.10	1.98	4.54	0.03	0.04
ST 5	1.29	2.59	0.96	0.03	0.20	1.91	6.71	*BDL	0.02
ST 6	4.83	4.10	25.94	0.02	0.04	1.73	3.71	0.02	0.08
ST 7	3.01	2.11	14.86	0.02	0.06	2.27	2.54	0.02	0.17
HT 2	1.25	0.41	*BDL	*BDL	0.12	1.24	4.07	*BDL	0.03
HT 4	1.72	8.47	5.98	0.02	0.70	46.20	194.96	0.06	0.47
HT 8	11.77	5.25	73.53	0.04	0.32	23.56	35.54	0.17	0.57
HSC 9	5.81	4.07	58.84	0.03	*BDL	4.22	7.22	0.03	0.20
HSC 28	2.46	4.10	14.37	0.02	0.11	4.21	21.38	0.04	0.12
HSC 30	5.27	3.08	8.35	0.02	0.12	3.81	10.59	0.03	0.33
HSC 33	3.00	10.91	6.85	0.02	0.32	1.48	28.59	*BDL	0.10
SANS ^31^	300	400	2000	NA	70	2000	5000	10	10

LOD—Limit of detection; *BDL -below detection limit; NA—Not available.

### Hazard quotient (HQ) and carcinogenic risk assessment

Table 9 presents the exposure dosage and hazard quotient (HQ) for ingestion and dermal pathway for metals. The HQ_ing_ and HQ_derm_ for all analyzed trace metals in both children and adults were less than one unit, indicating that there are no potential non-carcinogenic health risks associated with consumption of the water. Table 10 presents the total Hazard Quotient and Health risk index (HI) for trace metals in the water samples, showing that residents of the study area are not susceptible to non-cancer risks due to exposure to trace metals in drinking water. Table 11 presents the cancer risk associated with the levels of Ni, As and Pb in the drinking water samples. The table shows that only the maximum levels of lead had the highest chance of cancer risks for both adults and children.

**Table 9:** Reference dose (µg/kg/day) of metals, exposure dosage and hazard quotient (HQ) for ingestion and dermal pathway.

Metals	RFD_ing_	RFD_derm_	EXP_ing_	EXP_derm_	HQ_ing_	HQ_derm_	EXP_ing_	EXP_derm_	HQ_ing_	HQ_derm_
			Adult	Adult	Adult	Adult	Children	Children	Children	Children
Al (mean)	700	140	1.41E−01	7.30E−04	2.02E−04	5.21E−06	3.26E−01	4.89E−05	4.66E−04	3.49E−07
Minimum			3.62E−02	1.87E−04	5.17E−05	1.34E−06	8.36E−02	1.25E−05	1.19E−04	8.96E−08
Maximum			3.91E−01	2.02E−03	5.59E−04	1.45E−05	9.04E−01	1.36E−04	1.29E−03	9.68E−07
Mn (mean)	140	0.96	1.11E−01	5.74E−04	7.92E−04	5.98E−04	2.56E−01	3.84E−05	1.83E−03	4.00E−05
Minimum			1.18E−02	6.11E−05	8.44E−05	6.37E−05	2.73E−02	4.10E−06	1.95E−04	4.27E−06
Maximum			3.16E−01	1.64E−03	2.26E−03	1.71E−03	7.31E−01	1.10E−04	5.22E−03	1.14E−04
Fe (mean)	700	140	5.38E−01	2.78E−03	7.68E−04	1.99E−05	1.24E+00	1.86E−04	1.78E−03	1.33E−06
Minimum			2.79E−02	1.44E−04	3.98E−05	1.03E−06	6.43E−02	9.65E−06	9.19E−05	6.89E−08
Maximum			2.13E + 00	1.10E−02	3.05E−03	7.88E−05	4.93E+00	7.39E−04	7.04E−03	5.28E−06
Co (mean)	20	5.4	6.94E−04	7.13E−06	3.47E−05	1.32E−06	1.60E−03	4.78E−07	8.02E−05	8.85E−08
Minimum			5.38E−04	5.53E−06	2.69E−05	1.02E−06	1.24E−03	3.71E−07	6.22E−05	6.86E−08
Maximum			1.08E−03	1.11E−05	5.39E−05	2.05E−06	2.49E−03	7.42E−07	1.25E−04	1.37E−07
Ni (mean)	20	5.4	6.84E−03	7.03E−05	3.42E−04	1.30E−05	1.58E−02	4.71E−06	7.91E−04	8.73E−07
Minimum			1.02E−03	1.05E−05	5.10E−05	1.94E−06	2.36E−03	7.02E−07	1.18E−04	1.30E−07
Maximum			2.04E−02	2.10E−04	1.02E−03	3.88E−05	4.71E−02	1.40E−05	2.36E−03	2.60E−06
Cu (mean)	40	12	1.95E−01	1.01E−03	4.87E−03	8.39E−05	4.50E−01	6.75E−05	1.12E−02	5.62E−06
Minimum			3.61E−02	1.87E−04	9.02E−04	1.56E−05	8.34E−02	1.25E−05	2.08E−03	1.04E−06
Maximum			1.34E+00	6.93E−03	3.35E−02	5.78E−04	3.10E+00	4.64E−04	7.74E−02	3.87E−05
Zn (mean)	300	60	6.95E−01	2.15E−03	2.32E−03	3.58E−05	1.61E+00	1.44E−04	5.35E−03	2.40E−06
Minimum			7.36E−02	2.27E−04	2.45E−04	3.78E−06	1.70E−01	1.52E−05	5.67E−04	2.54E−07
Maximum			5.65E+00	1.74E−02	1.88E−02	2.91E−04	1.31E+01	1.17E−03	4.35E−02	1.95E−05
As (mean)	0.3	0.123	1.83E−03	9.47E−06	6.10E−03	7.70E−05	4.23E−03	6.35E−07	1.41E−02	5.16E−06
Minimum			6.42E−04	3.32E−06	2.14E−03	2.70E−05	1.48E−03	2.23E−07	4.94E−03	1.81E−06
Maximum			5.01E−03	2.59E−05	1.67E−02	2.11E−04	1.16E−02	1.74E−06	3.86E−02	1.41E−05
Pb (mean)	3.5	0.42	4.73E−03	9.75E−05	1.35E−03	2.32E−04	1.09E−02	6.51E−06	3.12E−03	1.55E−05
Minimum			6.41E−04	1.32E−05	1.83E−04	3.14E−05	1.48E−03	8.82E−07	4.23E−04	2.10E−06
Maximum			1.64E−02	3.38E−04	4.69E−03	8.05E−04	3.79E−02	2.26E−05	1.08E−02	5.38E−05

**Table 10:** Total Hazard Quotient and Health risk index (HI) for trace metals in the water samples.

Metals	Mean	Minimum HQ (Adult)	Maximum	Mean	Minimum HQ (Children)	Maximum
Al	2.07E−04	5.30E−05	5.73E−04	4.66E−04	1.20E−04	1.29E−03
Mn	1.39E−03	1.48E−04	3.97E−03	1.87E−03	1.99E−04	5.34E−03
Fe	7.88E−04	4.08E−05	3.13E−03	1.78E−03	9.20E−05	7.04E−03
Co	3.60E−05	2.79E−05	5.60E−05	8.03E−05	6.23E−05	1.25E−04
Ni	3.55E−04	5.30E−05	1.06E−03	7.91E−04	1.18E−04	2.36E−03
Cu	4.95E−03	9.18E−04	3.41E−02	1.13E−02	2.09E−03	7.74E−02
Zn	2.35E−03	2.49E−04	1.91E−02	5.36E−03	5.67E−04	4.36E−02
As	6.18E−03	2.17E−03	1.69E−02	1.41E−02	4.95E−03	3.86E−02
Pb	1.58E−03	2.15E−04	5.50E−03	3.14E−03	4.26E−04	1.09E−02
HI	1.78E−02	3.87E−03	8.44E−02	3.88E−02	8.62E−03	1.87E−01

**Table 11:** Cancer risk associated with the levels of Ni, As and Pb in the drinking water.

	SFing (µg /kg/day)	CRing (Adult)	CRing (Children)
Ni (Mean)	910	7.52E−06	1.74E−05
Minimum		1.12E−06	2.59E−06
Maximum		2.24E−05	5.18E−05
As (mean)	1500	1.22E−06	2.82E−06
Minimum		4.28E−07	9.89E−07
Maximum		3.34E−06	7.72E−06
Pb (mean)	8.5	5.57E−04	1.29E−03
Minimum		7.55E−05	1.74E−04
Maximum		1.93E−03	4.46E−03

## Discussion

This study provides information about the quality of drinking water in a selected rural community of Thulamela municipality of Limpopo province, South Africa, taking into consideration the physicochemical, microbiological and trace metals’ parameters of the treated water supplied to the village by the government, through the municipality. Many participants in the study have their primary source of water piped into their yards, while very few have water in their houses. This implies that getting water for household use would involve collecting the water from the yard and then into the storage containers. Those who do not have the taps in their yards have to collect water from the neighbours’ yards or the street taps. This observation is not restricted to the study area, as a similar situation has been observed in other rural communities of Limpopo Province^21^. This need to pass water through multiple containers before the point of use increases the risk of contamination.

Residents of the study area, just like residents of other settlements in Thulamela Municipality^21^, store their drinking water in plastic buckets, ceramic vessels, jerry cans and other containers. Almost all the respondents (96.5%) claim that their water storage vessels are covered and that their drinking water usually stays for less than a week in the storage containers (87.8%). Covering of water storage containers reduces the risk of water contamination from dust or other airborne particles. However, intermittent interruption of municipal water supply lasting for a week or more in the study area and the consequent use of alternative sources of water predispose the residents to various health risks as intermittent interruption in water supply has been linked to higher chances of contamination in the distribution systems, compared with continuous supply; in addition, the alternative sources of water may not be of a good quality as the treated municipal water^33,34^, yet, more than half of the respondents in this study (53%) use water directly from source without any form of treatment. This is because many residents in rural communities of Limpopo province believe that the water they drink is of good quality and thus do not need any further treatment^21^. The few who treat their water before drinking mostly use the boiling method. While boiling and other home-based interventions like solar disinfection of water have been reported to improve the quality of drinking water; drinking vessels, like cups, have also been implicated in water re-contamination of treated water at the point of use^16^ and most respondents (91.3%) in this study admittedly use cups to collect water from the storage containers. The risk of contamination is even increased when cups without handles are used, where there is a higher chance that the water collector would touch the water in the container with his/her fingers. The Centres for Disease Control and Prevention (CDC) recommends that containers for drinking water should be fitted with a small opening with a cover or a spigot, through which water can be collected while the container remains closed, without dipping any potentially contaminated object into the container^35^. However, it is noteworthy that all the respondents claim to always wash their hands with soap (or its equivalents) and water after using the toilets, a constant practice of hand washing after using the toilet has been associated with a reduced risk of water contamination with *E. coli*^19^.

Treated water from the dam tested negative for both total coliform and *E. coli* hence complied with regulatory standards of SANS^31^ and WHO^8^. The results could probably be due to the use of chlorine as a disinfectant in the treatment plant. Using disinfectants, pathogenic bacteria from the water can be killed and water made safe for the user. Similar studies have also reported that treated water in urban water treatment plants contains no total coliforms and *E. coli*^36^. In contrast, treated water sources in rural areas have been reported to have considerable levels of total coliform and *E. coli*^37^. The reason alluded to this include lack of disinfectant, no residual chlorine in the treated water, high prevalence of open defecation and unhygienic practices in proximity to water sources^38^.

From the water samples collected from the street taps, 62.5% were found to be contaminated with total coliform during the dry season, while the percentage rose to 87.5% during the wet season. The street tap which is about 13 km from the reservoir recorded high levels of total coliform ranging from 1.0 -2000 MPN/100 mL with most of the sites exceeding the WHO guidelines of 10 MPN/100 mL^8^. In both seasons, all the samples tested negative for *E. coli*, this complies with the WHO guideline of 0 MPN/100 mL. While the water leaving the treatment plant met bacteriological standards, the detection of coliform bacteria in the distribution lines suggest that the water is contaminated in the distribution networks. This could be due to the adherence of bacteria onto biofilms or accidental point source contamination by broken pipes, installation and repair works^39^. Furthermore, the water samples from households’ storage containers were contaminated by total coliform (73% and 85%) and *E. coli* (10.4% and 13.2%) during the dry and wet season, respectively. Microbiological contamination of household water stored in containers could be due to unhygienic practices occurring between the collection point and the point-of-use^40,41^.

Generally, higher levels of contamination were recorded in the wet season than in the dry season. The wet season in Thulamela Municipality is often characterized with increased temperature which could lead to favourable condition for microbial growth. Also, the treatment plant usually makes use of the same amount of chlorine for water purification during both seasons, even though influent water would be of a higher turbidity during the wet season, hence reducing the levels of residual chlorine^42^.

The pH of the analyzed samples from the study area ranged from 7.15 to 9.92. Most of the samples were within the values recommended by SANS (5 to 9.7) and comparable to results from previous similar studies^31,43^. Also, the electrical conductivity of all water samples from this study ranged from 28 µS/cm to 903 µS/cm which complied with the recommended value of SANS: < 1700 µS/cm^31^. The presence of dissolved solids such as calcium, chloride, and magnesium in water samples is responsible for its electrical conductivity^44^.

Total dissolved solids are the inorganic salts and small amounts of organic substance, which are present as solution in water^45^. Water has the ability to dissolve a wide range of inorganic and some organic minerals or salts such as potassium, calcium, sodium, bicarbonates, chlorides, magnesium, sulphates, etc. These minerals produced unwanted taste and colour in water^46^. A high TDS value indicates that water is highly mineralised. The recommended TDS value set for drinking water quality is ≤ 1200 mg/L^31^. In this study, the TDS values ranged from 18 mg/L to 572 mg/L. Hence, the TDS of all the household’s storage samples complied with the guidelines and consistent with previous studies^47^.

The analysis of magnesium (1.32 to 16.59 mg/L) and calcium (2.14 to 31.65 mg/L) concentrations showed that they were within the permissible range recommended for drinking water by SANS^31^ and WHO^8^. All living organisms depend on magnesium in all types of cells, body tissues and organs for variety of functions while calcium is very important for human cell physiology and bones. Similar studies in Ethiopia and Turkey also showed acceptable levels of these metals in drinking water^46,48^. Likewise, the levels of potassium (0.14 to 1.85 mg/L) and sodium (0.18 to 12.96 mg/L) were within the permissible limit of WHO and SANS and may not cause health related problems. Sodium is essential in humans for the regulation of body fluid and electrolytes, and for proper functioning of the nerves and muscles, however, excessive sodium in the body can increase the risk of developing a high blood pressure, cardiovascular diseases and kidney damage^49,50^. Potassium is very important for protein synthesis and carbohydrate metabolism, thus, it is very important for normal growth and body building in humans, but, excessive quantity of potassium in the body (hyperkalemia) is characterized with irritability, decreased urine production and cardiac arrest^51^.

Metals like copper (Cu), cobalt (Co) and zinc (Zn) are essential requirements for normal body growth and functions of living organisms, however, in high concentrations, they are considered highly toxic for human and aquatic life^42^. Elevated trace metal(loids) concentrations could deteriorate water quality and pose significant health risks to the public due to their toxicity, persistence, and bio accumulative nature^52^. In this study, the concentrations of Manganese, Cobalt, Nickel and Copper all complied with the recommended concentration by SANS for domestic water use.

Aluminum concentration in the drinking water samples ranged from 1.25—13.46 µg/L. All analysed samples complied with the recommended concentration of ≤ 300 µg/L for domestic water use^31^. The recorded levels of Al in water from this study should not pose any health risk. At a high concentration, aluminium affects the nervous system, and it is linked to several diseases, such as Parkinson’s and Alzheimer’s diseases^53^. Iron (Fe) is an essential element for human health, required for the production of protein haemoglobin, which carries oxygen from our lungs to the other parts of the body. Insufficient or excess levels of iron can have negative effect on body functions^54^. The recommended concentration of iron in drinking water is ≤ 2000 µg/L^31^. In this study, the concentration of iron in the samples ranged from 0.96 to 73.53 µg/L. Similar results were reported by Jamshaid et al. in Khyber Pakhtunkhwa province^55^. A high concentration of Fe in water can give water a metallic taste, even though it is still safe to drink^56^.

The levels of Pb, As and Zn were in the range of 0.02–0.57 µg/L, 0.02–0.17 µg/L, and 2.54–194.96 µg/L, respectively whereas Cr was not detected in the samples collected. The levels recorded complied with the SANS^31^ and WHO^8^ guidelines for drinking water. Similar results were reported by Mohod and Dhote^57^. Lead is not desirable in drinking water because it is carcinogenic and can cause growth impairment in children^41^. Inorganic arsenic is a confirmed carcinogen and is the most significant chemical contaminant in drinking-water globally^44^. Zinc deficiency can cause loss of appetite, decreased sense of taste and smell, slow wound healing and skin sores^58^. Cr is desirable at low concentration but can be harmful if present in elevated levels.

The hazard quotient (HQ) takes into consideration the oral toxicity reference dose for a trace metal that humans can be exposed to^59^. Health related risk associated with the exposure through ingestion depends on the weight, age and volume of water consumed by an individual. HQ_ing_ and HQ_derm_ for all analyzed trace metals in both children and adults were less than one unit (Table 9), indicating that there are no potential non-carcinogenic health risks associated with the consumption of the water from the study area either by children or adults. The calculated average cumulative health risk index (HI) for children and adult was 3.88E-02 and 1.78E-02, respectively. HQ across metals serve as a conservative assessment tool to estimate high-end risk rather than low end-risk in order to protect the public. This served as a screen value to determine whether there is major significant health risk^60^. The results in this study signifies that the population of the investigated area are not susceptible to non-cancer risks due to exposure to trace metals in drinking water. Similar observation has been reported by Bamuwamye et al. after investigating human health risk assessment of trace metals in Kampala (Uganda) drinking water^61^. It should be noted that the hazard index values for children were higher than that of adult, suggesting that children were more susceptible to non-carcinogenic risk from the trace metals.

Drinking water with trace metals such as Pb, As, Cr and Cd could potentially enhance the risk of cancer in human beings^62,63^. Long term exposure to low amounts of toxic metals might, consequently, result in many types of cancers. Using As, Ni and Pb carcinogens, the total exposure risks of the residents in Table 11. For trace metals, an acceptable carcinogenic risk value of less than 1 × 10^−6^ is considered as insignificant and the cancer risk can be neglected; while an acceptable carcinogenic risk value of above 1 × 10^–4^ is considered as harmful and the cancer risk is worrisome. Amongst the studied trace metals, only the maximum levels of lead for both adults and children had the highest chance of cancer risks (1.93E−03 and 4.46E−03) while Arsenic and Nickel have no chance of cancer risk with values of 3.34E−06; 7.72E−06 and 2.24E−05; 5.18E−05, in both adults and children respectively. The only cancer risk to residents of the studied area could be from the cumulative ingestion of lead in their drinking water. The levels of Pb recorded in this study complied to the SANS guideline value for safe drinking water. While the levels of Pb from the dam and the street pipes were relatively low, higher levels where recorded at household taps and storage containers and this may be due to the kind of storage containers and pipes used in those households. Generally, the water supply is of low Pb levels which should not pose any health risk to the consumers. However, the residents in rural areas should be properly educated on the kind of materials to be used for safe storage of water which should not pose an additional health burden. The likelihood of cancer risk was only associated with the consumption of the highest levels of Pb reported for a life time for adults (set at 70 years) and 6 years for children. Consistent consumption of water from the same source throughout an adult’s lifetime is unlikely as residents in those communities may change their locations at some points, hence reducing the possible risk associated with consistent exposure to the same levels of Pb.

## Conclusions

The study shows that as distance increases from the treatment reservoir to distribution points, the cross-contamination rate also increases, therefore, good hygienic practices is required while transporting, storing and using water. Unhygienic handling practices at any point between collection and use contribute to the deterioration of drinking water quality.

The physicochemical, bacteriological quality and trace metals’ concentration of water samples from treated source, street taps and household storage containers were majorly within the permissible range of both WHO and SANS drinking water standards. HQ for both children and adults were less than unity, showing that the drinking water poses less significance health threat to both children and adults. Amongst the studied trace metals, only the maximum level of lead for both adults and children has the highest chance of cancer risks.

We recommend that appropriate measures should be taken to maintain residual free chlorine at the distribution points, supply of municipal treated water should be more consistent in all the rural communities of Thulamela municipality, Limpopo province and residents should be trained on hygienic practices of transportation and storage of drinking water from the source to the point of use.

## Data Availability

The datasets used and analysed during the current study are available from the first author on reasonable request.

## Abbreviations

APHA: American Public Health Association
CDC: Centres for Disease Control and Prevention
D: Dam
DWAF: Department of Water Affairs and Forestry
EC: Electrical conductivity
HI: Health risk index
HQ: Hazard quotient
HSC: Household storage containers
HT: Household taps
ICP-MS: Inductively coupled plasma mass spectrophotometer
ICP-OES: Inductively coupled plasma optical emission spectrophotometer
MPN: Most probable number
SANS: South African National Standards
ST: Street taps
TDS: Total Dissolved Solids
UNGA: United Nations General Assembly
UNICEF: United Nations International Children Emergency Fund
USEPA: United States Environmental Protection Agency
WHO: World Health Organization

## References

[1] Taiwo, A.M., Olujimi, O.O., Bamgbose, O. & Arowolo, T.A. Surface water quality monitoring in Nigeria: Situational analysis and future management strategy. In *Water Quality Monitoring and Assessment* (ed. Voudouris, K) 301–320 (IntechOpen, 2012).

[2] Corcoran, E., *et al.* Sick water? The central role of wastewater management in sustainable development: A rapid response assessment. United Nations Enviromental Programme UN-HABITAT, GRID-Arendal. https://wedocs.unep.org/20.500.11822/9156 (2010).

[3] United Nations, The 2030 Agenda and the Sustainable Development Goals: An opportunity for Latin America and the Caribbean (LC/G.2681-P/Rev.3), Santiago (2018).

[4] Hubert E, Wolkersdorfer C (2015). Establishing a conversion factor between electrical conductivity and total dissolved solids in South African mine waters. Water S.A..

[5] Department of Water Affairs (DWA). Groundwater Strategy. Department of Water Affairs: Pretoria, South Africa. 64 (2010).

[6] Lu Y, Nakicenovic N, Visbeck M, Stevance AS (2015). Policy: Five priorities for the UN sustainable development goals. Nature.

[7] Shaheed A, Orgil J, Montgomery MA, Jeuland MA, Brown J (2014). Why, “improved” water sources are not always safe. Bull. World Health Organ..

[8] WHO. *Guidelines for Drinking Water Quality* 4th Edn (World Health Organization, Geneva, Switzerland, 2011). http://apps.who.int/iris/bitstream/10665/44584/1/9789241548151_eng.pdf.

[9] Patil PN, Sawant DV, Deshmukh RN (2012). Physico-chemical parameters for testing of water—a review. Int. J. Environ. Sci..

[10] Bain R, Cronk R, Wright J, Yang H, Slaymaker T, Bartram J (2014). Fecal contamination of drinking-water in low-and middle-income countries: A systematic review and meta-analysis. PLoS Med..

[11] Younos, T. & Grady, C.A. Potable water, emerging global problems and solutions. In *The Handbook of Environmental Chemistry* 30 (2014).

[12] Tigabu AD, Nicholson CF, Collick AS, Steenhuis TS (2013). Determinants of household participation in the management of rural water supply systems: A case from Ethiopia. Water Policy..

[13] Oljira, G. Investigation of drinking water quality from source to point of distribution: The case of Gimbi Town, in Oromia Regional State of Ethiopia (2015).

[14] Clasen T, Haller L, Walker D, Bartram J, Cairncross S (2007). Cost-effectiveness of water quality interventions for preventing diarrhoeal disease in developing countries. J. Water Health.

[15] Too JK, Sang WK, Ng’ang’a Z, Ngayo MO (2016). Fecal contamination of drinking water in Kericho District, Western Kenya: Role of source and household water handling and hygiene practices. J. Water Health.

[16] Rufener S, Mausezahl D, Mosler H, Weingartner R (2010). Quality of drinking-water at source and point-of-consumption—drinking cup as a high potential recontamination risk: A field Study in Bolivia. J. Health Popul. Nutri..

[17] Nsubuga FNW, Namutebi EN, Nsubuga-ssenfuma M (2014). Water resources of Uganda: An assessment and review. Water Resour. Prot..

[18] Rawway M, Kamel MS, Abdul-raouf UM (2016). Microbial and physico-chemical assessment of water quality of the river Nile at Assiut Governorate (Upper Egypt). J. Ecol. Health Environ..

[19] Agensi, A., Tibyangye, J., Tamale, A., Agwu, E. & Amongi C. Contamination potentials of household water handling and storage practices in Kirundo Subcounty, Kisoro District, Uganda. *J. Environ. Public Health.* Article ID 7932193, 8 pages (2019). 10.1155/2019/7932193 PMC6421758 30944573

[20] Mahmud ZH (2019). Occurrence of *Escherichia coli* and faecal coliforms in drinking water at source and household point-of-use in Rohingya camps, Bangladesh. Gut Pathog..

[21] Edokpayi JN (2018). Challenges to sustainable safe drinking water: A case study of water quality and use across seasons in rural communities in Limpopo Province, South Africa. Water.

[22] Musyoki A, Thifhulufhelwi R, Murungweni FM (2016). The impact of and responses to flooding in Thulamela Municipality, Limpopo Province, South Africa, Jàmbá. J. Disaster Risk Stud..

[23] Census 2011. Main Place: Lufule. Accessed from Census 2011: Main Place: Lufule (adrianfrith.com) on 30/01/2022.

[24] Bolarinwa OA (2015). Principles and methods of validity and reliability testing of questionnaires used in social and health science researches. Niger. Postgrad. Med. J..

[25] Association APH (1992). Standard Methods for the Examination of Water and Waste Water.

[26] Bernardes C, Bernardes R, Zimmer C, Dorea CC (2020). A simple off-grid incubator for microbiological water quality analysis. Water.

[27] Rich CR, Sellers JM, Taylor HB, IDEXX Laboratories Inc. Chemical reagent test slide. U.S. Patent Application 29/218,589. (2006).

[28] Naveedullah (2014). Concentrations and human health risk assessment of selected heavy metals in surface water of siling reservoir watershed in Zhejiang Province, China. Pol. J. Environ. Stud..

[29] Wu J, Sun Z (2016). Evaluation of shallow groundwater contamination and associated human health risk in an alluvial plain impacted by agricultural and industrial activities, mid-west China. Expos. Health..

[30] Wu L, Zhang X, Ju H (2007). Amperometric glucose sensor based on catalytic reduction of dissolved oxygen at soluble carbon nanofiber. Biosens. Bioelectron..

[31] South African National Standard (SANS). 241-1: Drinking Water, Part 1: Microbiological, Physical, Aesthetic and Chemical Determinants. 241-2: 2015 Drinking Water, Part 2: Application of SANS 241-1 (2015).

[32] Department of Water Affairs and Forestry (DWAF). South African Water Quality Guidelines (second edition). Volume 1: Domestic Use, (1996).

[33] Drake, M.J. & Stimpfl, M. Water matters. In *Lunar and Planetary Science Conference*, Vol. 38, 1179 (2007).

[34] Kumpel E, Nelson KL (2016). Intermittent water supply: prevalence, practice, and microbial water quality. Environ. Sci. Technol..

[35] Centers for Disease Control and Prevention. The safe water system: Safe storage of drinking water. Accessed from CDC Fact Sheet on 30/01/2022 (2012).

[36] Hashmi I, Farooq S, Qaiser S (2009). Chlorination and water quality monitoring within a public drinking water supply in Rawalpindi Cantt (Westridge and Tench) area, Pakistan. Environ. Monit. Assess..

[37] Onyango, A. E., Okoth, M. W., Kunyanga, C. N. & Aliwa, B. O. Microbiological quality and contamination level of water sources in Isiolo country in Kenya. *J. Environ. Public Health*. **2018**, 2139867 (2018). 10.1155/2018/2139867 PMC6077557 30112010

[38] Gwimbi P, George M, Ramphalile M (2019). Bacterial contamination of drinking water sources in rural villages of Mohale Basin, Lesotho: Exposures through neighbourhood sanitation and hygiene practices. Environ. Health Prev. Med..

[39] Karikari AY, Ampofo JA (2013). Chlorine treatment effectiveness and physico-chemical and bacteriological characteristics of treated water supplies in distribution networks of Accra-Tema Metropolis, Ghana. Appl. Water Sci..

[40] Thompson T, Sobsey M, Bartram J (2003). Providing clean water, keeping water clean: An integrated approach. Int. J. Environ. Health Res..

[41] Cronin AA, Breslin N, Gibson J, Pedley S (2006). Monitoring source and domestic water quality in parallel with sanitary risk identification in Northern Mozambique to Prioritise protection interventions. J. Water Health.

[42] Edokpayi JN, Enitan AM, Mutileni N, Odiyo JO (2018). Evaluation of water quality and human risk assessment due to heavy metals in groundwater around Muledane area of Vhembe District, Limpopo Province, South Africa. Chem. Cent. J..

[43] Edimeh PO, Eneji IS, Oketunde OF, Sha’Ato R (2011). Physico-chemical parameters and some heavy metals content of Rivers Inachalo and Niger in Idah, Kogi State. J. Chem. Soc. Nigeria.

[44] Rahmanian, N., *et al*. Analysis of physiochemical parameters to evaluate the drinking water quality in the State of Perak, Malaysia. *J. Chem.* 1–10. Article ID 716125 (2015).

[45] WHO/FAO (2003). Diet, Nutrition, and the Prevention of Chronic Diseases.

[46] Meride Y, Ayenew B (2016). Drinking water quality assessment and its effects on resident’s health in Wondo genet campus, Ethiopia. Environ. Syst. Res..

[47] Mapoma HW, Xie X (2014). Basement and alluvial aquifers of Malawi: An overview of groundwater quality and policies. Afr. J. Environ. Sci. Technol..

[48] Soylak M, Aydin F, Saracoglu S, Elci L, Dogan M (2002). Chemical analysis of drinking water samples from Yozgat. Turkey Pol. J. Environ. Stud..

[49] Munteanu C, Iliuta A (2011). The role of sodium in the body. Balneo Res. J..

[50] Strazzullo P, Leclercq C (2014). Sodium. Adv. Nutr..

[51] Pohl HR, Wheeler JS, Murray HE (2013). Sodium and potassium in health and disease. Met. Ions Life Sci..

[52] Muhammad S, Shah MT, Khan S (2011). Health risk assessment of heavy metals and their source apportionment in drinking water of Kohistan region, northern Pakistan. Microchem. J..

[53] Inan-Eroglu E, Ayaz A (2018). Is aluminum exposure a risk factor for neurological disorders?. J. Res. Med. Sci..

[54] Milman N (2008). Prepartum anaemia: Prevention and treatment. Ann. Hematol..

[55] Jamshaid M, Khan AA, Ahmed K, Saleem M (2018). Heavy metal in drinking water its effect on human health and its treatment techniques—a review. Int. J. Biosci..

[56] Tagliabue A, Aumont O, Bopp L (2014). The impact of different external sources of iron on the global carbon cycle. Geophys. Res. Lett..

[57] Mohod CV, Dhote J (2013). Review of heavy metals in drinking water and their effect on human health. Int. J. Innov. Res. Technol. Sci. Eng..

[58] Bhowmik D, Chiranjib KP, Kumar S (2010). A potential medicinal importance of zinc in human health and chronic. Int. J. Pharm..

[59] Mahmud MA (2017). Low temperature processed ZnO thin film as electron transport layer for efficient perovskite solar cells. Sol. Energy Mater. Sol. Cells..

[60] Rajan S, Ishak NS (2017). Estimation of target hazard quotients and potential health risks for metals by consumption of shrimp (*Litopenaeus vannamei*) in Selangor, Malaysia. Sains Malays..

[61] Bamuwamye M (2017). Human health risk assessment of heavy metals in Kampala (Uganda) drinking water. J. Food Res..

[62] Saleh HN (2019). Carcinogenic and non-carcinogenic risk assessment of heavy metals in groundwater wells in Neyshabur Plain, Iran. Biol. Trace Elem. Res..

[63] Tani FH, Barrington S (2005). Zinc and copper uptake by plants under two transpiration rates Part II Buckwheat (*Fagopyrum esculentum* L.). Environ. Pollut..

